# Psychometric evaluation of the Chinese version of the Educational Leadership Scale for Nursing Students: a quantitative cross-sectional design

**DOI:** 10.3389/fpsyg.2025.1604416

**Published:** 2025-11-12

**Authors:** Xiaodong Yu, Xu Wang, Xiuli Wang

**Affiliations:** 1 Neurointerventional Ward, Department of Vascular Surgery, The First Affiliated Hospital of Jinzhou Medical University, Jinzhou, China; 2 Department of Emergency Surgery, The First Affiliated Hospital of Jinzhou Medical University, Jinzhou, China; 3 Cancer Clinical Research Ward, The First Affiliated Hospital of Jinzhou Medical University, Jinzhou, China

**Keywords:** educational leadership, nursing education, nursing students, psychometrics, leadership

## Abstract

**Objective:**

The Educational Leadership Scale for Nursing Students (ELS-NS) was introduced and revised as part of this study, which also aimed to evaluate the scale’s validity and reliability in a Chinese cultural setting.

**Methods:**

This study was a cross-sectional study conducted from March to October 2024. The research employed a multifaceted approach that included internal consistency testing, item analysis, exploratory factor analysis, and confirmatory factor analysis. The sample comprised 508 nursing students from three medical universities located in Liaoning Province, China. Furthermore, a subset of 50 nursing students participated in a retest after a two-week interval to evaluate the reliability of the findings.

**Results:**

The Chinese version of the ELS-NS comprised 19 items, and exploratory factor analysis revealed three distinct factors that accounted for 67.251% of the total variance. Confirmatory factor analysis confirmed the adequacy of a three-factor model, as evidenced by the following fit indices: *χ*^2^/*df* = 0.112, RMSEA = 0.015, SRMR = 0.034, CFI = 0.997, TLI = 0.997, AGFI = 0.958 and PGFI = 0.758. This study conducted a comprehensive assessment of the reliability and validity of the Chinese version of the ELS-NS, with all indicators meeting psychometric standards. Firstly, in terms of reliability, the scale demonstrates excellent stability and internal consistency. The Cronbach’s *α* coefficient for the total scale was 0.940, with Cronbach’s α coefficients for each dimension ranging from 0.841 to 0.943, and the composite reliability (CR) was above 0.7. The split-half reliability for the Chinese version of the ELS-NS was 0.764; the retest reliability was 0.926. Secondly, in terms of validity, the scale also demonstrates good measurement validity. The content validity index (CVI) of the scale was 0.84. The factor loadings for each item exceeded 0.6, the average variance extracted (AVE) was greater than 0.5, and the correlation coefficients were lower than the square root of the AVE, indicating robust convergent and discriminant validity.

**Conclusion:**

The Chinese adaptation of the Educational Leadership Scale for Nursing Students (ELS-NS) has been demonstrated to be a valid and reliable instrument for evaluating nursing students’ inclination toward educational leadership across three dimensions: visionary leadership, instructional leadership, and scientific leadership. This assessment contributes to the enhancement of nursing education and practice by providing insights from various perspectives.

## Introduction

Leadership as a multidimensional composite concept involves both individual ability trait elements but also involves teamwork mechanisms and ultimately points to the strategic realization of organizational goals (Ford et al., 2016). Leadership is of particular value in the education and health professions, where educators use it to build a vision for development, optimize resource allocation, and inspire teams, and health professionals use it to enhance service effectiveness and drive professional innovation (Nieuwboer et al., 2019). Especially in the field of nursing specialization, contemporary nursing practice places higher demands on nurses’ complex competencies, i.e., nurses not only need to possess clinical expertise but also need to assume multiple leadership roles such as educational guidance, team coordination, and quality control (Goldsberry, 2018).

Nursing education leadership is an important dimension of professional competence development; its formation mechanism has distinctive disciplinary characteristics and stage-by-stage development law (Flores et al., 2022). Domestic and international studies have shown that modern nursing education leaders need to effectively integrate multidimensional elements such as organizational structure optimization, cultural environment creation, and interpersonal network construction in the dynamically evolving healthcare ecosystem to achieve continuous improvement of nursing quality through systematic educational leadership behaviors (Cummings et al., 2008; Cummings et al., 2021; Steinert et al., 2012; Wong et al., 2024). Previously, the internationally commonly used tool for measuring nursing education leadership was *the* Nursing Education Leadership Assessment Scale *(NELAS).* This scale constructs a 32-item assessment system across 6 dimensions, including educational decision-making, team coordination, and resource integration, and has been widely applied in the field of nursing education in multiple European and American countries (Smith et al., 2019; Garcia et al., 2020). The validation results in the Spanish nursing education population showed that the correlation coefficients between the scale’s dimensions ranged from 0.42 to 0.68. The criterion-related validity was significantly positively correlated with the scores of the *Nurse Manager Competency Scale* (r = 0.73, *p* < 0.001) (Garcia et al., 2020). Additionally, in a validation study conducted in South Korea (Lee et al., 2022), the scale was found to yield significantly different leadership assessments for nursing educators with varying teaching experience (< 5 years, 5–10 years, > 10 years) (*F* = 4.27, *p* = 0.01), with those having longer teaching experience scoring higher in the “Educational Planning” and “Clinical Mentorship” dimensions. However, it should be noted that there is currently a lack of standardized leadership assessment tools suitable for the local nursing education context in China. Additionally, direct use of the original version of the scale is associated with issues such as insufficient cultural adaptability and potential deviations in language comprehension. The concept of full-cycle training should be established for the cultivation of such professional leadership ability, and the foundational role of the professional education stage should be particularly emphasized (Kim et al., 2022). Through systematically designed curriculum and clinical practice, nursing students can gradually build a framework of core competencies such as decision-making judgment, interdisciplinary communication, teamwork, etc., so as to lay a foundation of competencies for their future roles as clinical educators, quality supervisors, and discipline leaders (Akdeniz and Duygulu, 2024; Aydogdu, 2023). Although the current research field of nursing education in China has formed a consensus perception of leadership development, there is still a significant disconnect between theory and practice at the level of empirical research. A systematic literature analysis revealed a clear structural imbalance in existing research findings: 84.6% of empirical studies focused on the leadership characteristics of working nurses, while the development of research tools specific to the nursing student population lagged significantly (Ystaas et al., 2023). This bias in research orientation directly constrains the scientific construction of the leadership development system in nursing education and leads to a lack of an accurate assessment basis for educational interventions. Based on the competency development theory perspective, a model of professionalism for nursing educators should include three key dimensions: judgment of teaching situations, integration of educational theories, and translation into clinical practice (Atalla et al., 2024; De Juan Pardo et al., 2022; Liu, 2024). The theoretical framework of educational leadership further explains that effective instructional coaching requires a dynamic balance of three dimensions: adherence to scientific principles, innovation in educational strategies, and foresight in developmental planning, which, through synergy, form the core of the effectiveness of educational leadership (Maqbool et al., 2023; Keshmiri, 2023; Bacon et al., 2023). It is of concern that a standardized leadership assessment system based on Bloom’s Taxonomy of Educational Objectives has not yet been established in the current field of international nursing education. As a classic educational assessment framework, Bloom’s Taxonomy provides multidimensional theoretical coordinates for the assessment of educational leadership through the hierarchical constructs of the cognitive domain (knowledge, comprehension, application, analysis, synthesis, and evaluation) and the affective domain (attitudes and values) (Im et al., 2018; Stephens and Ormandy, 2019; Davy et al., 2025). In particular, the high degree of fit between its higher-order dimensions of the cognitive domain and the elements of leadership provides an important theoretical reference for the development of assessment tools with clinical contextual adaptations (Miles and Scott, 2019). Future research is urgently needed to break through the traditional flat model of competency assessment and construct a three-dimensional assessment model based on three dimensions (cognitive hierarchy, emotional development, and behavioral performance), which will become a key breakthrough to optimize the pathway of leadership development in nursing education (Mohammed and Omar, 2020).

The advancement of nursing education in China has increasingly underscored the importance of fostering comprehensive literacy and leadership competencies among nursing students. To facilitate targeted educational interventions, there is a pressing need for scientifically sound and effective assessment instruments to evaluate the educational leadership capabilities of nursing students. The Educational Leadership Scale for Nursing Students (ELS-NS), developed by Turkish scholar Karaman, is grounded in Bloom’s Taxonomy of Educational Objectives and specifically addresses the unique requirements of the nursing student demographic. This scale has exhibited strong reliability and validity within the Turkish nursing student population (Karaman F. et al., 2023; Karaman S. et al., 2023). The present study aims to introduce and culturally adapt the ELS-NS for application within the Chinese nursing student population, thereby assessing its reliability and validity in this context. The implementation of this assessment tool is anticipated to enhance the comprehensiveness and diversity of evaluations in nursing education and practice, ultimately contributing to the elevation of nursing education quality from multiple perspectives. Furthermore, it serves as a significant reference for fostering teamwork and communication in nursing practice, thereby improving the quality and efficiency of nursing services.

## Methods

### Study design and selection of participants

This study was a cross-sectional study conducted from March to October 2024. The research comprehensively assessed nursing students’ competence in educational leadership in three areas: scientific leadership, instructional leadership, and visionary leadership. Based on the design principles of international scales (Rockstuhl and Van Dyne, 2018) and our prior research experience, the sample size should be 5 to 10 times the number of items. With 19 items in this study, the required sample size ranged from 95 to 190 cases. The total sample size for this study was 508 cases. Through convenience sampling, 508 nursing students were recruited from three medical universities in Shenyang, Jinzhou, and Dalian, Liaoning Province, China, to participate in this study to ensure the robustness of the exploratory and validation factor analyses.

Inclusion criteria for subjects: (1) aged ≥18 years; (2) currently enrolled in full-time nursing programs at three medical universities in Liaoning Province; (3) voluntarily agreed to participate; (4) ability to effectively complete the questionnaire.

Exclusion criteria for subjects: students with severe physical illnesses (such as cardiovascular disease or immune system disorders), mental health conditions (such as diagnosed depression or anxiety disorders), or those who are pregnant may be excluded due to the potential impact of these conditions on their ability to consistently participate in the study or on data quality.

### Measurements

#### General demographics questionnaire

The research team developed a general demographic questionnaire for subjects who met the inclusion criteria. The questionnaire consisted of seven items: gender, age, education level, place of residence, whether they had experience as student leaders, whether they voluntarily chose to specialize in nursing, and whether they had received management-related courses and skills training.

#### Educational leadership scale for nursing students

The instrument was developed by Karaman F. et al. (2023) and Karaman S. et al. (2023) to assess the potential for educational leadership among nursing students from three distinct perspectives: scientific leadership, instructional leadership, and visionary leadership. This scale comprises 19 items categorized into three dimensions, utilizing a five-point Likert scale (where 5 represents “strongly agree,” 4 indicates “somewhat agree,” 3 denotes “neutral,” 2 signifies “disagree,” and 1 corresponds to “strongly disagree”). The total scores obtainable from the scale range from 19 to 95, with higher scores reflecting a greater inclination toward educational leadership among nursing students. The overall Cronbach’s alpha coefficient for the scale was determined to be 0.92, indicating a high level of reliability.

### Processes

#### Data collection process

Following standardized training, the researchers were organized into three groups, each consisting of four members, and subsequently recruited nursing students from three medical universities located in Liaoning Province. Initially, 50 nursing students were chosen to participate in a preliminary survey utilizing a post-translation scale, which was then administered again 2 weeks later to assess the retest reliability of the instrument. For the primary survey, a total of 534 students were initially selected; however, 26 responses were deemed invalid, yielding a final count of 508 valid questionnaires for analysis.

#### Process for translation and cross-cultural adaptation

Upon receiving authorization from Professor Funda via email, the scale underwent translation following Brislin’s model (Khalaila, 2013). Initially, two professors proficient in both Chinese and English translated the scale into Chinese, resulting in what is referred to as edition A. This version was subsequently refined through collaborative efforts between the researchers and the professors, leading to the creation of edition B. Following this, two native English-speaking professors, who had no prior exposure to the original scale, performed a back-translation of edition B into English. After further deliberations, edition C was established. The researchers and translators then conducted a comparative analysis of edition C against the original scale to finalize the first draft of the Chinese Educational Leadership Scale for Nursing Students. To ensure both content validity and cross-cultural adaptation, nine experts in nursing education and psychometrics were consulted (Kim et al., 2022). These experts were tasked with integrating their theoretical knowledge with clinical experience to suggest modifications aimed at enhancing the clarity and comprehensibility of the scale items, while taking into account their cultural context, applicability, and relevance. This methodology was implemented to ensure the cultural appropriateness and content equivalence of the questionnaire. Simultaneously, the relevance of each item in version C to the study’s objectives was assessed and rated using a 4-point Likert scale, which facilitated the measurement of the scale’s content validity. The researcher compiled and organized the feedback from the consultations, and based on the experts’ recommendations, the group engaged in discussions to revise the scale’s content, ultimately resulting in version D.

#### Process of data analysis

The results were evaluated using SPSS version 25.0 and AMOS version 24.0. The validity of the scale was tested by using the content validity index. The structural validity, the aggregation validity and the discrimination validity were evaluated based on the scores from the second round of expert consultation [Exploratory factor analysis (*EFA*) was conducted on 254 questionnaires, while another 254 questionnaires were used for confirmatory factor analysis (*CFA*)]. All statistical tests were conducted as two-sided, with a significance threshold set at a *p* value of less than 0.05.

#### Analysis of item

The item analysis employed the critical ratio method, which involved categorizing the overall scores of 508 students into two distinct groups: the top 27% (high subgroup) and the bottom 27% (low subgroup). Differences between these groups were assessed using independent sample t-tests. Furthermore, Pearson correlation analysis was performed to investigate the relationship between individual item scores and the total scale score. The established criteria included (Zhang et al., 2022): (1) a critical ratio (*CR*) exceeding 3.0 for each item; and (2) a correlation coefficient greater than 0.4 between item scores and the total scale score.

#### Analysis of reliability

The scale’s internal consistency was assessed through the Cronbach’s *α* coefficient and the Spearman-Brown split-half reliability coefficient. To determine the scale’s stability, the test–retest reliability coefficient was applied. The criteria set for these assessments were: (1) the Cronbach’s α for the scale and its components should be at least 0.7; (2) the split-half reliability coefficient should be at least 0.6; and (3) the test–retest reliability coefficient should be at least 0.7.

#### Analysis of validity

To evaluate content validity, a panel of nine specialists in nursing education was assembled to assess each item on the scale, categorizing them as irrelevant, somewhat relevant, relevant, or highly relevant. Items classified as irrelevant or somewhat relevant were assigned a score of 0, while those deemed relevant or highly relevant received a score of 1. The Item-Content Validity Index (*I-CVI*) was computed as the ratio of specialists who rated an item as 1 to the total number of specialists. The overall content validity score for the scale was derived by calculating the average of all *I-CVIs*. A minimum *I-CVI* threshold of ≥0.7 and a Scale-Content Validity Index (*S-CVI*) of ≥ 0.9 were established as criteria for validity. The factor structure of the scale was examined through *EFA* and *CFA*. The Kaiser-Meyer-Olkin (*KMO*) measure and Bartlett’s test of sphericity were utilized in the *EFA*, with a requisite *KMO* value exceeding 0.60 and a significant Bartlett’s test (*p* < 0.05 and *CR* > 0.7) indicating high validity (Mikkonen et al., 2017). Discriminant validity was assessed by comparing the square root of the Average Variance Extracted (*AVE*) with the correlation coefficients of observable variables. A greater square root of *AVE* than the correlation coefficient signifies stronger discriminant validity (Wu et al., 2022).

### Approval of ethics

Our study followed the ethical guidelines set forth in the 1964 Declaration of Helsinki (Holt, 2014). We obtained informed consent from all participants, who filled out anonymous questionnaires. This research received approval from the Ethics Committee of Jinzhou Medical University (JZMULL2024080).

## Results

### Demographic characteristics

In this study, a survey was conducted with 508 nursing students, including 23 (4.5%) males and 485 (95.5%) females, the majority of participants are predominantly within the age range of 18 to 29 years, with a mean age of 21.72 years and a standard deviation of 2.79, more than half (67.1%) of the nursing students had received bachelor’s degree education, and 76.8% (390) of the nursing students were from towns. There were 128 (25.2%) nursing students who had experience as student leaders; 394 (77.6%) nursing students who voluntarily chose nursing as their major; 64.4% (327) of the nursing students had received relevant courses and skills training in management; as detailed within Table 1.

**Table 1:** Frequency distribution of demographic characteristics (*n* = 508).

Factors	Group	*n*	%
Sex	Male	23	4.5%
	Female	485	95.5%
Education level	College and below	101	19.9%
	Bachelor	341	67.1%
	Master	66	13.0%
Place of residence	City	390	76.8%
	Countryside	118	23.2%
Have there been any experiences as a student leader?	Yes	128	25.2%
	No	380	74.8%
Have the nursing major been chosen voluntarily?	Yes	394	77.6%
	No	114	22.4%
Have you received any theoretical coursework or skills training related to management?	Yes	327	64.4%
	No	181	35.6%

### Translation and intercultural adaptation

Before the formal survey, the research group conducted a cultural survey and a pre-survey to obtain preliminary feedback. After obtaining the results of these surveys, the team organized members to have in-depth discussions on the scale entries, systematically sorted out the scale entries according to the situation reflected in the surveys, and precisely and meticulously modified and improved the entries that did not meet the requirements or had ambiguities. According to the Chinese cultural background, we followed four principles of modification, and the following are the specific modification process and explanation of the wording of the dimensions and entries:

(1) Terminology localization: to replace westernized or directly translated terms with terms commonly used in the Chinese education/healthcare system; for example, amend entry 10, “Criteria for National Core Nursing Programs,” to read “National Guidelines for Nursing Education”; and amend entry 11, “Criteria for International Nursing Education Programs,” to read “General Norms for International Nursing Education.”
(2) Normalization of expression: i.e., adopting sentence structures that are more natural in the Chinese context; for example, adjusting entry 12, “Nursing education courses should be designed in a way that facilitates learning,” to “Nursing courses should be designed in accordance with the laws of learning”; adjust entry 5, “Teaching materials should be designed according to the learning objectives of nursing students,” to “The design of teaching materials should be oriented to the learning objectives of nursing students”; adjustment of entry 7, “Selection of the most appropriate method to determine the training needs of nursing students,” to “Assessment of the training needs of nursing students using scientific methods.”
(3) Adaptation of values, i.e., reflecting the collectivism, practical orientation and ethical requirements emphasized in Chinese education; for example, adjusting entry 15, “Introducing professional ethics is important,” to “Strengthening education on professional ethics”; adjustment of entry 16, “Design of learning modules based on nursing ethics,” to “Integration within ethics cases into course modules”; adjustment of entry 18, “Cooperation skills,” to “Teamwork skills”; adjust entry 13, “Use of nursing techniques,” to “Integration with clinical practice techniques.”
(4) Avoiding sensitive words: weakening political expressions (e.g., “democracy”) that may lead to ambiguity; for example, replacing entry 19, “democratic atmosphere,” with “equal and interactive teaching atmosphere”; adjusting entry 17, “communication skills,” with “two-way communication skills of teachers and students.”

The scale underwent a validation process that included cultural adaptation and a preliminary survey, during which no items were removed or added. The preliminary survey indicated that the questionnaire required approximately 5–10 min for completion. The finalized Chinese version of the Educational Leadership Scale for Nursing Students comprises three dimensions—visionary leadership, instructional leadership, and scientific leadership—consisting of 19 items that are systematically organized and tailored to align with the local cultural context.

### Analysis of item

The scores from the Chinese version of the Nursing Student Educational Leadership Scale were divided into high and low subgroups, with the top 27% (≥54 points) classified as high and the bottom 27% (≤34 points) classified as low. An independent samples *t*-test was conducted to evaluate each item in the high and low groups, yielding *t*-values ranging from 11.043 to 26.684 (all *p* < 0.001). Additionally, Pearson correlation analysis revealed correlation coefficients between each item and the total score of the scale ranging from 0.546 to 0.786 (all *p* < 0.001). No items were removed, as indicated in Table 2.

**Table 2:** Item analysis for Chinese version of the Educational Leadership Scale for Nursing Students (*n* = 508).

Item	Correlation coefficient between item and total score	High grouping	Low grouping	Critical ratio	*P*
ELS-NS 1	0.769	3.96 ± 0.99	1.70 ± 0.70	22.050	<0.001
ELS-NS 2	0.711	4.14 ± 0.78	1.67 ± 0.80	25.950	<0.001
ELS-NS 3	0.713	3.70 ± 0.90	1.68 ± 0.74	20.450	<0.001
ELS-NS 4	0.769	3.90 ± 0.97	1.62 ± 0.60	23.517	<0.001
ELS-NS 5	0.753	3.93 ± 0.95	1.67 ± 0.65	22.996	<0.001
ELS-NS 6	0.786	3.90 ± 0.99	1.53 ± 0.63	23.577	<0.001
ELS-NS 7	0.686	3.64 ± 1.13	1.73 ± 0.68	17.026	<0.001
ELS-NS 8	0.785	4.07 ± 0.87	1.58 ± 0.66	26.684	<0.001
ELS-NS 9	0.753	4.07 ± 0.98	1.62 ± 0.72	23.633	<0.001
ELS-NS 10	0.756	3.88 ± 0.83	1.57 ± 0.71	24.758	<0.001
ELS-NS 11	0.681	3.62 ± 1.34	1.60 ± 0.69	15.802	<0.001
ELS-NS 12	0.626	3.33 ± 1.31	1.63 ± 0.71	13.455	<0.001
ELS-NS 13	0.619	3.34 ± 1.29	1.79 ± 0.77	12.152	<0.001
ELS-NS 14	0.654	3.53 ± 1.22	1.67 ± 0.74	15.329	<0.001
ELS-NS 15	0.668	3.50 ± 1.29	1.58 ± 0.67	15.502	<0.001
ELS-NS 16	0.546	3.33 ± 1.11	1.65 ± 0.79	14.505	<0.001
ELS-NS 17	0.654	3.53 ± 1.26	1.67 ± 0.74	14.909	<0.001
ELS-NS 18	0.643	3.54 ± 1.31	1.65 ± 0.66	15.067	<0.001
ELS-NS 19	0.576	3.39 ± 1.39	1.90 ± 0.77	11.043	<0.001

### Analysis of reliability

The Chinese adaptation of the Nursing Student Educational Leadership Scale demonstrated a Cronbach’s alpha coefficient of 0.940, with dimensional coefficients of 0.943, 0.884, and 0.841, respectively. Additionally, a Spearman-Brown split-half coefficient of 0.764 was recorded, alongside dimensional split-half coefficients of 0.943, 0.882, and 0.845. Furthermore, a test–retest reliability coefficient of 0.926 was established from a sample of 50 participants who were reassessed 2 weeks later, as detailed in Table 3.

**Table 3:** Reliability analysis of the Chinese version of the Educational Leadership Scale for Nursing Students.

The scale and its dimension	Cronbach′s alpha	Split-half reliability	Test–retest reliability
ELS-NS	0.940	0.764	0.926
Visionary leadership	0.943	0.943	_
Instructional leadership	0.884	0.882	_
Scientific leadership	0.841	0.845	_

### Analysis of validity

#### Analysis of content validity

Nine specialists evaluated the content validity of the Chinese versions of ELS-NS. As shown in Table 4, the I-CVI ranged from 0.78 to 1.00, the S-CVI/UA was 0.84, and the S-CVI/AVe was 0.95.

**Table 4:** Content validity analysis of the Chinese version of the Educational Leadership Scale for Nursing Students.

Item	Experts (score)									I-CVI
	1	2	3	4	5	6	7	8	9	
ELS-NS 1	1	1	1	1	1	1	1	1	1	1.000
ELS-NS 2	1	1	1	1	1	1	1	1	1	1.000
ELS-NS 3	1	1	1	1	1	1	1	1	1	1.000
ELS-NS 4	1	1	1	1	0	1	1	1	1	0.889
ELS-NS 5	1	1	1	1	1	1	1	1	1	1.000
ELS-NS 6	1	1	1	1	1	1	1	1	1	1.000
ELS-NS 7	1	1	1	1	1	1	1	1	1	1.000
ELS-NS 8	1	1	1	1	1	1	1	1	1	1.000
ELS-NS 9	1	0	1	1	1	1	1	1	1	0.889
ELS-NS 10	1	1	1	1	1	1	1	0	1	0.889
ELS-NS 11	1	1	1	1	1	1	1	1	1	1.000
ELS-NS 12	1	1	1	1	1	1	1	1	1	1.000
ELS-NS 13	1	1	1	1	1	1	1	1	1	1.000
ELS-NS 14	1	1	1	1	1	1	1	1	1	1.000
ELS-NS 15	1	1	1	1	1	1	1	1	1	1.000
ELS-NS 16	1	1	1	1	1	1	1	1	1	1.000
ELS-NS 17	1	1	1	0	1	1	1	1	1	0.889
ELS-NS 18	1	1	1	1	1	1	1	0	1	0.889
ELS-NS 19	1	1	1	1	1	1	1	1	1	1.000

#### Exploratory factor analysis

**Figure 1:**
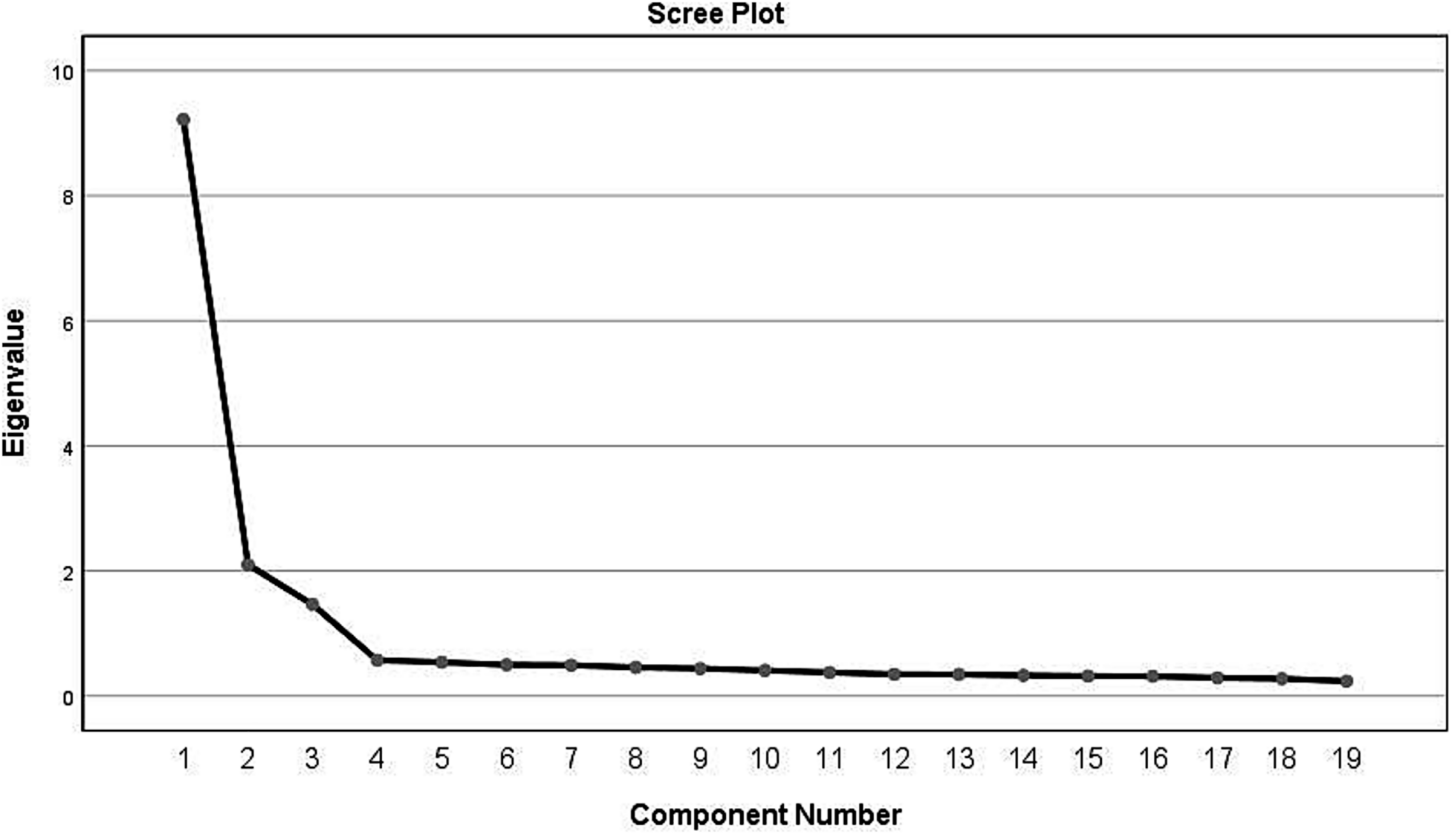
Scree plot of exploratory factor analysis for the Chinese version of the Educational Leadership Scale for Nursing Students.

The initial set of 254 questionnaires was employed for exploratory factor analysis. The Chinese version of the ELS-NS demonstrated a Kaiser-Meyer-Olkin (KMO) measure of 0.959, while Bartlett’s test of sphericity produced an approximate chi-square value of 6129.431 (*p* < 0.001), thereby affirming its appropriateness for factor analysis. Principal Component Analysis (PCA) revealed three potential factors, each exhibiting an initial eigenvalue exceeding 1. As illustrated in Table 5 and supported by the fragmentation plot in Figure 1, this analysis indicated the absence of multifactor loadings. Collectively, these factors accounted for 67.251% of the total variance. Based on the content characteristics of these common factors, they were classified as visionary leadership, instructional leadership, and scientific leadership.

**Table 5:** Factor loading from exploratory factor analysis of the Chinese version of the Educational Leadership Scale for Nursing Students.

Item	Factor 1	Factor 2	Factor 3
ELS-NS 10	**0.783**		–
ELS-NS 11	**0.722**		–
ELS-NS 12	**0.764**		–
ELS-NS 13	**0.777**		–
ELS-NS 14	**0.799**		–
ELS-NS 15	**0.782**		–
ELS-NS 16	**0.706**		–
ELS-NS 17	**0.823**		–
ELS-NS 18	**0.750**	–	
ELS-NS 19	**0.751**	–	–
ELS-NS 5	–	**0.799**	–
ELS-NS 6	–	**0.744**	–
ELS-NS7	–	**0.750**	–
ELS-NS 8	–	**0.757**	–
ELS-NS 9	–	**0.803**	–
ELS-NS 1	–	–	**0.756**
ELS-NS 2	–	–	**0.775**
ELS-NS 3	–	–	**0.769**
ELS-NS 4	–	–	**0.759**
Eigenvalue	9.218	2.098	1.462
Cumulative variance contribution	33.448	52.191	67.251

Bolded numbers indicate that the factor loading for that item on the corresponding factor is ≥0.5, while loadings on all other factors are <0.4, satisfying the requirements for convergent and discriminant validity in factor analysis.

#### Confirmatory factor analysis

**Figure 2:**
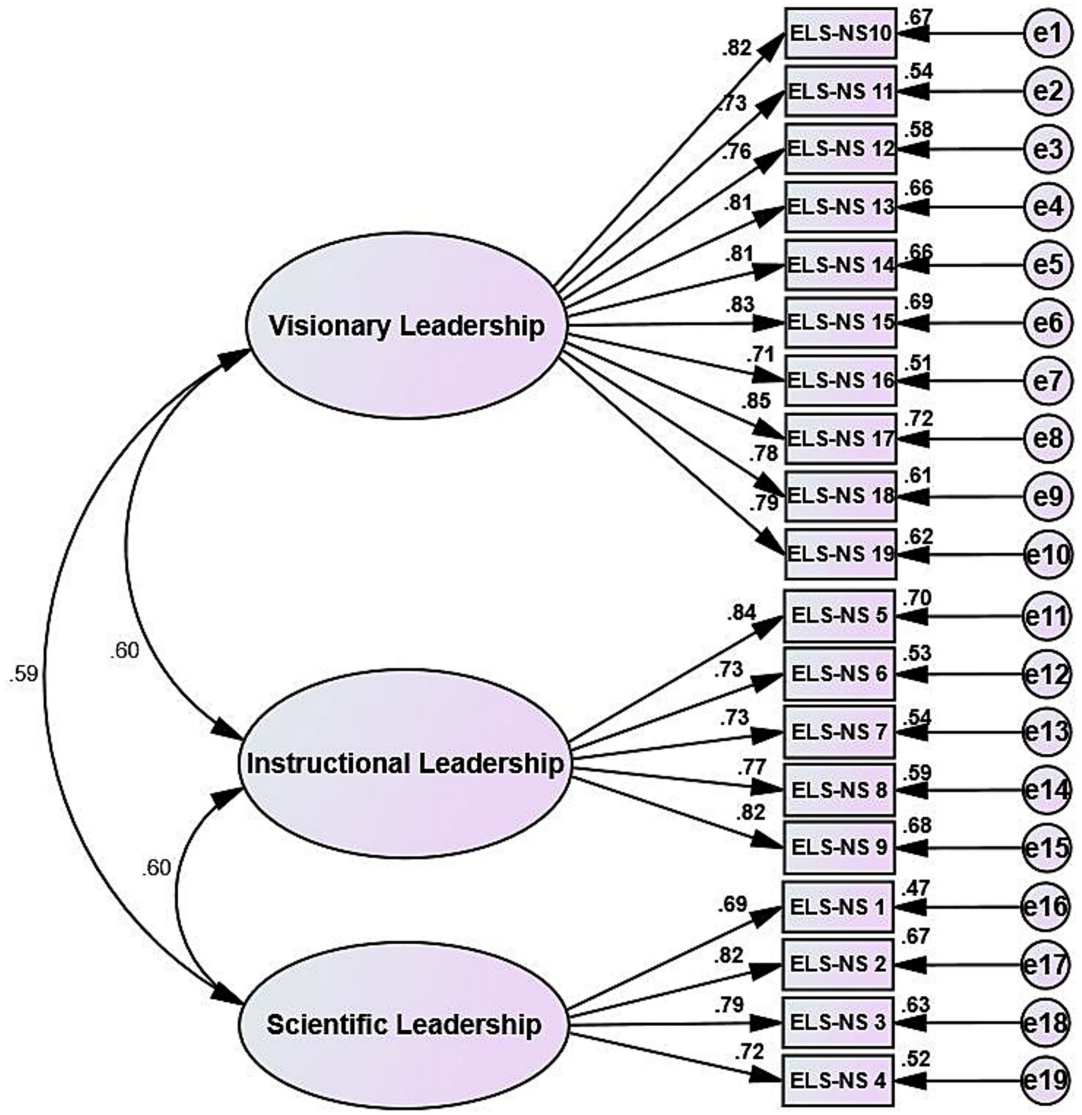
Standardized three-factor structural model of the Chinese version of the Educational Leadership Scale for Nursing Students.

A total of 254 remaining questionnaires were used for confirmatory factor analysis. A three-factor structural model was constructed using AMOS 24.0 and analyzed using maximum likelihood estimation. The final model fit indices were as follows: *χ*^2^/*df* = 0.112(≦3), RMSEA = 0.015(<0.05), SRMR = 0.034(<0.05), CFI = 0.997(≥0.95), TLI = 0.997(≥0.95), AGFI = 0.958(≥0.9) and PGFI = 0.758(>0.7), as shown in Figure 2. Referencing the research by Wolf and McNeish (2023), it is concluded that all key indicators in this study meet their respective established threshold ranges. All indicators have reached or even significantly exceeded their respective excellence standards, indicating that the three-factor model exhibits excellent data fit and possesses strong construct validity. This provides robust support for the validity and reliability of the scale.

#### Convergent validity

Table 6 illustrates that all factor loadings are greater than 0.6, the average variance extracted (*AVE*) exceeds 0.5, and the composite reliability (*CR*) is above 0.7. These findings collectively affirm the presence of robust convergent validity.

**Table 6:** Convergent validity.

Item	Factor	Estimate	AVE	CR
ELS-NS 1	Factor 3	0.688	0.572	0.842
ELS-NS 2	Factor 3	0.891		
ELS-NS 3	Factor 3	0.792		
ELS-NS 4	Factor 3	0.720		
ELS-NS 5	Factor 2	0.822	0.607	0.885
ELS-NS 6	Factor 2	0.769		
ELS-NS7	Factor 2	0.732		
ELS-NS 8	Factor 2	0.730		
ELS-NS 9	Factor 2	0.835		
ELS-NS 10	Factor 1	0.787	0.625	0.943
ELS-NS 11	Factor 1	0.783		
ELS-NS 12	Factor 1	0.851		
ELS-NS 13	Factor 1	0.715		
ELS-NS 14	Factor 1	0.828		
ELS-NS 15	Factor 1	0.810		
ELS-NS 16	Factor 1	0.811		
ELS-NS 17	Factor 1	0.760		
ELS-NS 18	Factor 1	0.734		
ELS-NS 19	Factor 1	0.816		

#### Discriminant validity

According to the classic criterion proposed by Fornell and Larcker in 1981 (Fornell and Larcker, 1981), discriminant validity is established when the correlation coefficient between any two latent variables (factors) is less than the square root of their respective AVE values. This study satisfies this condition, indicating that the factors maintain significant correlations while possessing sufficient discriminant power. Notable correlations were identified among factors 1, 2, and 3 (*p* < 0.001), as presented in Table 7. All correlation coefficients were found to be less than the square root of the average variance extracted (*AVE*), thereby demonstrating robust discriminant validity.

**Table 7:** Discriminant validity.

Factor	F1	F2	F3
F1	0.625	–	–
F2	0.598***	0.607	–
F3	0.591***	0.597***	0.572
Square root of AVE	0.791	0.779	0.757

The values along the diagonal represent the Average Variance Extracted (AVE), while the triangular values beneath indicate the Pearson correlation coefficients among the dimensions, with all *p*-values being less than 0.001.

## Discussion

The objective of this research was to translate the Educational Leadership Scale for Nursing Students (ELS-NS) into Chinese and to conduct a cross-cultural adaptation of the instrument. The translation process strictly followed the Brislin model, and expert consultation was used to ensure contextual adaptation of the scale (Li et al., 2023). Nine domain experts revised the initial translation to fit the localized context of educational leadership development and assessment of nursing students in China. In the initial survey, 50 nursing students recognized the simplicity, clarity, and contextual relevance of the Chinese version of the ELS-NS and provided feedback on the comprehensibility of the scale structure and wording. The completed Chinese version of the ELS-NS comprises 19 items organized into three dimensions. An item analysis revealed that the scale items were successfully differentiated from one another (Xu et al., 2024), with moderate to high correlations observed between each item and the overall scale. Reliability assessment indicated that the Cronbach’s alpha coefficient for the Chinese version of the scale remained consistent with that of the original version, suggesting that it possesses strong reliability and discriminant validity.

The evaluation of internal consistency and test–retest reliability is essential for determining the reliability of the scale (Anselmi et al., 2019). The Cronbach’s alpha coefficient for the Chinese adaptation of the ELS-NS was 0.940 (dimension-specific: 0.841–0.943), marginally higher than the original version (Karaman F. et al., 2023; Karaman S. et al., 2023) to indicate enhanced internal consistency. A split-half reliability coefficient of 0.764 further supported this, while test–retest reliability (assessing temporal stability; Leppink and Pérez-Fuster, 2017) was 0.926 for the Chinese ELS-NS, confirming its stability over time and overall reliability. This research assessed the content validity of the Chinese ELS-NS via nine experts: the Item-Level Content Validity Index (I-CVI) ranged from 0.78 to 1.00, and the Scale-Level Content Validity Index/Universal Agreement (S-CVI/UA) was 0.84, both exceeding established thresholds (Curtis and Keeler, 2021) to confirm contextual consistency. Exploratory Factor Analysis (EFA) identified three factors (accounting for 67.251% of total variance, item loadings > 0.5), consistent with the original scale’s structure (Karaman et al., 2023); Confirmatory Factor Analysis (CFA) further validated the three-factor model’s fit, together affirming structural validity. Each dimension’s Average Variance Extracted (AVE) > 0.5 and Composite Reliability (CR) > 0.7 supported robust convergent validity (Jung and Yang, 2022). Moreover, interdimensional correlation coefficients were lower than the square root of AVE, confirming strong discriminant validity (Fernandes et al., 2024) by clearly delineating the three attributes and minimizing confounding variables.

In a nutshell, the Chinese-adapted version of the Educational Leadership Skills Scale for Nursing Students (ELS-NS) has demonstrated validity for nursing education research and application in terms of both content and structure—consistent with prior validation studies. The original 19-item, three-factor ELS-NS (visionary, instructional, and scientific leadership) developed with Turkish nursing students showed acceptable psychometric properties, including Cronbach’s *α* > 0.70 for all dimensions and sufficient model fit via factor analysis (Karaman S. et al., 2023). A subsequent Persian adaptation further confirmed this three-factor structure (explaining 63.80% of total variance) with strong internal consistency (Cronbach’s *α* = 0.924) (Safari et al., 2024). Compared to these, the Chinese version maintains the three-factor framework while exhibiting marginally enhanced performance: its overall Cronbach’s α (0.940) and dimension-specific coefficients (0.841–0.943) exceed both the original and Persian versions, with a higher cumulative variance explanation rate (67.251% vs. 63.80% in Persian) (Karaman S. et al., 2023; Safari et al., 2024).

Like its predecessors, the Chinese ELS-NS explores leadership tendency differences across the three dimensions. By using this validated tool to foster nursing students’ educational leadership (as supported by cross-cultural evidence), students can better lead nursing discipline development, boost academic/professional influence, and improve team/patient outcomes (Abdelaziz et al., 2024). Nursing’s nature demands such leadership dispositions, which per ELS-NS studies promote professional growth, communication skills, and nursing practice innovation.

First, visionary leadership emphasizes the nursing student’s ability to see and strategically plan for trends in the nursing discipline. Contemporary nursing practice requires practitioners to anticipate dynamic changes in the healthcare ecosystem and develop adaptive strategies in advance. The Chinese version of the ELS-NS quantified nursing students’ potential in this dimension through the entries of “anticipating future nursing needs” and “setting long-term developmental goals.” The results of this study echo Malashenko et al. (2023) concept of “full-cycle training,” suggesting that nursing education needs to train nursing students in strategic thinking at an early stage to prepare them for their future roles as leaders in their disciplines. Second, instructional leadership focused on the knowledge transfer and team coordination effectiveness of nursing students as educators (Lyu et al., 2024). The scale items, such as “the design of teaching materials should be oriented to the learning objectives of nursing students” and “professional ethics cases should be integrated into the course modules,” reflect the practice-oriented and collectivist values emphasized in Chinese nursing education. Nursing educators need to have the ability to judge teaching situations and integrate educational theories, and the ELS-NS provides a precise basis for educational interventions by evaluating nursing students’ abilities in instructional design, teacher-student communication (Li et al., 2024), and so on. In addition, this study showed that only 25.2% of nursing students had experience as student leaders, suggesting that the development of instructional leadership skills needs to be combined with diverse practical experiences and strengthen the integration of clinical and teaching scenarios. Finally, scientific leadership embodies nursing students’ core ability to optimize nursing decisions using scientific methods. The Chinese version of the Educational Leadership Scale for Nursing Students (ELS-NS) further highlights the pivotal role of data-driven decision-making in the dimension of scientific leadership through item designs such as “using scientific methods to assess nursing students’ training needs” and “integrating clinical practice techniques with scientific decision-making.” As a core element driving the continuous improvement of nursing quality, the cultivation of scientific leadership is closely linked to systematic educational design—data from this study show that 64.4% of nursing students have received training in management courses. This result not only provides empirical support for the “promoting role of curriculum systems in enhancing scientific literacy” but also indirectly reflects the emphasis of current nursing education on fostering the basic competencies required for scientific leadership. However, this study has limitations due to restricted research conditions: a single sample source and insufficient representation. Future research should expand the sample size, collect data from more medical schools nationwide to further validate the scale’s applicability and reliability, and explore its utility in evaluating leadership training program effectiveness or correlating with nursing students’ long-term professional outcomes.

## Conclusion

The introduction and cultural adaptation of the Educational Leadership Scale for Nursing Students (ELS-NS) in China has been completed, confirming its psychometric stability in different educational contexts. The scale constructs a scientific nursing education quality assessment system by systematically quantifying the comprehensive competencies of educators (especially nursing students and junior nurses) in the three core dimensions of strategic planning, teaching practice, and research leadership. It provides a multidimensional competency development framework for nursing educators, promotes the in-depth integration of educational leadership and professional training goals, and facilitates the high-quality development of the nursing discipline. It should be noted that this study was conducted based on survey data collected at a specific time point. Future research could adopt a longitudinal follow-up design to further explore the dynamic development patterns of nursing students’ scientific leadership and the long-term impact of management course training on the cultivation of scientific leadership. Meanwhile, in terms of data collection, objective assessment indicators (e.g., course assessment results, records of participation in scientific research practices) could be integrated in the future to complement self-reported data, thereby providing a more comprehensive perspective for the assessment of scientific leadership.

## Data Availability

The original contributions presented in the study are included in the article/Supplementary material, further inquiries can be directed to the corresponding author.

## References

[ref1] Abdelaziz M. Ibrahim N. Elsaid A. (2024). Transforming nursing education: the power of educational leadership in optimizing time management and competency. A review article. Nurse Educ. Today 132:105892. doi: 10.1016/j.nedt.2024.105892

[ref2] Akdeniz C. Duygulu S. (2024). Systematic review on characteristics and effects of leadership development interventions for nursing students. Nurse Educ. 49, E147–E152. doi: 10.1097/NNE.0000000000001540, PMID: 37994497

[ref3] Anselmi P. Colledani D. Robusto E. (2019). A comparison of classical and modern measures of internal consistency. Front. Psychol. 10:2714. doi: 10.3389/fpsyg.2019.02714, PMID: 31866905 PMC6904350

[ref4] Atalla A. D. G. Mostafa W. H. Ali M. S. S. (2024). Inspiring nurses' sustainability mindset: exploring the mediating role of organizational culture on the relationship between pro-social leader behaviors and nurses' sustainability consciousness. BMC Nurs. 23:675. doi: 10.1186/s12912-024-02314-z, PMID: 39322967 PMC11426114

[ref5] Aydogdu A. L. F. (2023). Perceptions of nursing students about leadership: a qualitative study. Nurse Educ. Today 128:105891. doi: 10.1016/j.nedt.2023.105891, PMID: 37393652

[ref6] Bacon C. T. Bailey K. D. Caramanica L. Losty L. S. Nelson-Brantley H. Prestia A. . (2023). Leadership science in nursing: creating new solutions for new challenges. J. Nurs. Adm. 53, 127–129. doi: 10.1097/NNA.0000000000001256, PMID: 36821495

[ref7] Cummings G. Lee H. Macgregor T. Davey M. Wong C. Paul L. . (2008). Factors contributing to nursing leadership: a systematic review. J. Health Serv. Res. Policy 13, 240–248. doi: 10.1258/jhsrp.2008.007154, PMID: 18806183

[ref8] Cummings G. G. Lee S. Tate K. Penconek T. Micaroni S. P. M. Paananen T. . (2021). The essentials of nursing leadership: a systematic review of factors and educational interventions influencing nursing leadership. Int. J. Nurs. Stud. 115:103842. doi: 10.1016/j.ijnurstu.2020.103842, PMID: 33383271

[ref9] Curtis A. C. Keeler C. (2021). Measurement in nursing research. Am. J. Nurs. 121, 56–60. doi: 10.1097/01.NAJ.0000753668.78872.0f, PMID: 34009166

[ref10] Davy C. Windle A. Marshall A. Harvey G. (2025). Leading the way: implementing aged care innovations. JBI Evid Implement. 23, 119–130. doi: 10.1097/XEB.0000000000000466, PMID: 39291725

[ref11] De Juan Pardo M. Á. Vissandjee B. Guillaumet Olives M. Cerezuela Torre M. Á. Gallart Fernández-Puebla A. (2022). Enhancing perceived leadership of nursing students through a student-led dedicated education unit in a community setting: a feasibility study. J. Prof. Nurs. 43, 152–161. doi: 10.1016/j.profnurs.2022.10.009, PMID: 36496239

[ref12] Fernandes C. S. Campos M. J. Moreira M. T. Lima A. Ferreira S. Martins M. M. (2024). Development and validation of the serious educational game in nursing appraisal scale. Nurs Report 14, 1148–1157. doi: 10.3390/nursrep14020087, PMID: 38804420 PMC11130971

[ref13] Flores C. A. D. S. Maier S. R. O. Moura A. A. Balsanelli A. P. Dias B. M. Bernardes A. (2022). Authentic leadership in the educational system and in nursing education: an integrative review. Rev. Bras. Enferm. 75:e20220122. doi: 10.1590/0034-7167-2022-0122, PMID: 36134769

[ref14] Ford K. Menchine M. Burner E. Arora S. Inaba K. Demetriades D. . (2016). Leadership and teamwork in trauma and resuscitation. West. J. Emerg. Med. 17, 549–556. doi: 10.5811/westjem.2016.7.29812, PMID: 27625718 PMC5017838

[ref15] Fornell C. Larcker D. F. (1981). Evaluating structural equation models with unobservable variables and measurement error. J. Mark. Res. 18, 39–50. doi: 10.1177/002224378101800104

[ref16] Garcia M. L. Rodriguez S. M. Martinez P. A. (2020). Cross-cultural adaptation of the NELAS: application in American baccalaureate nursing programs. Nurse Educ. Today 87:104421. doi: 10.1016/j.nedt.2020.104421

[ref17] Goldsberry J. W. (2018). Advanced practice nurses leading the way: interprofessional collaboration. Nurse Educ. Today 65, 1–3. doi: 10.1016/j.nedt.2018.02.024, PMID: 29518668

[ref18] Holt G. R. (2014). Declaration of Helsinki-the world's document of conscience and responsibility. South. Med. J. 107:407. doi: 10.14423/SMJ.0000000000000131, PMID: 25010578

[ref19] Im E. O. Broome M. E. Inouye J. Kunaviktikul W. Oh E. G. Sakashita R. . (2018). An emerging integrated middle-range theory on Asian women's leadership in nursing. J. Transcult Nurs. 29, 318–325. doi: 10.1177/1043659618760397, PMID: 29478380

[ref20] Jung H. Yang Y. (2022). Reliability and validity of the Korean version of the social justice scale in nursing students. Int. J. Environ. Res. Public Health 19:14443. doi: 10.3390/ijerph192114443, PMID: 36361322 PMC9659084

[ref21] Karaman F. KavgaoĞlu D. Yildirim G. Rashidi M. Ünsal Jafarov G. Zahoor H. . (2023). Development of the educational leadership scale for nursing students: a methodological study. BMC Nurs. 22:110. doi: 10.1186/s12912-023-01254-4, PMID: 37032331 PMC10084681

[ref22] Karaman S. Yilmaz Z. Kaya S. (2023). Development of the educational leadership scale for nursing students: a methodological study. A methodological research. J. Nurs. Educ. Pract. 13, 112–121. doi: 10.5430/jnep.v13n8p112 PMC10084681 37032331

[ref23] Keshmiri F. (2023). A developmental pathway toward leadership for educational change: the educators' experiences of the educational scholar program. BMC Med. Educ. 23:30. doi: 10.1186/s12909-023-04015-8, PMID: 36647074 PMC9843881

[ref24] Khalaila R. (2013). Translation of questionnaires into Arabic in cross-cultural research: techniques and equivalence issues. J. Transcult. Nurs. 24, 363–370. doi: 10.1177/1043659613493440, PMID: 23835895

[ref25] Kim H. O. Lee I. Lee B. S. (2022). Nursing leaders' perceptions of the state of nursing leadership and the need for nursing leadership education reform: a qualitative content analysis from South Korea. J. Nurs. Manag. 30, 2216–2226. doi: 10.1111/jonm.13596, PMID: 35301786 PMC10078751

[ref26] Lee H. J. Park S. Y. Kim M. K. (2022). Psychometric validation of the Korean version of the nursing education leadership assessment scale (NELAS) and its association with teaching experience. Nurse Educ. Today 114:105589. doi: 10.1016/j.nedt.2022.105589

[ref27] Leppink J. Pérez-Fuster P. (2017). We need more replication research – a case for test-retest reliability. Perspect. Med. Educ. 6, 158–164. doi: 10.1007/s40037-017-0347-z, PMID: 28390030 PMC5466566

[ref28] Li Y. Guo L. L. Gui J. Zhang X. Wang Y. Liu H. . (2024). Construction and application of “organ-system-centered” undergraduate nursing professional training model. BMC Nurs. 23:608. doi: 10.1186/s12912-024-02235-x, PMID: 39218861 PMC11367839

[ref29] Li J. Yang Z. Qi R. Tan M. Ji X. Hou B. . (2023). Psychometric evaluation of the Chinese version of motivation for nursing student scale (MNSS): a quantitative and cross-sectional design. Nurse Educ. Pract. 71:103690. doi: 10.1016/j.nepr.2023.103690, PMID: 37429219

[ref30] Liu H. Y. (2024). Effects of the design thinking pedagogy on design thinking competence of nursing students in Taiwan: a prospective non-randomized study. Nurse Educ. Today 138:106197. doi: 10.1016/j.nedt.2024.106197, PMID: 38636188

[ref31] Lyu X. Dong L. Fan Z. Sun Y. Zhang X. Liu N. . (2024). Artificial intelligence-based graded training of pulmonary nodules for junior radiology residents and medical imaging students. BMC Med. Educ. 24:740. doi: 10.1186/s12909-024-05723-5, PMID: 38982410 PMC11234785

[ref32] Maqbool S. Zafeer H. M. I. Zeng P. Mohammad T. Khassawneh O. Wu L. (2023). The role of diverse leadership styles in teaching to sustain academic excellence at secondary level. Front. Psychol. 13:1096151. doi: 10.3389/fpsyg.2022.1096151, PMID: 36698584 PMC9869245

[ref0020] Malashenko G. T. Kosov M. E. Frumina S. V. Grishina O. A. Alandarov R. A. Ponkratov V. V. . (2023). A Digital Model of Full-Cycle Training Based on the Zettelkasten and Interval Repetition System. Emerging Science Journal, 7, 1–15. doi: 10.28991/ESJ-2023-SIED2-01

[ref33] Mikkonen K. Elo S. Miettunen J. Saarikoski M. Kääriäinen M. (2017). Development and testing of the CALDs and CLES+T scales for international nursing students' clinical learning environments. J. Adv. Nurs. 73, 1997–2011. doi: 10.1111/jan.13268, PMID: 28152229

[ref34] Miles J. M. Scott E. S. (2019). A new leadership development model for nursing education. J. Prof. Nurs. 35, 5–11. doi: 10.1016/j.profnurs.2018.09.009, PMID: 30709465

[ref35] Mohammed M. Omar N. (2020). Question classification based on bloom's taxonomy cognitive domain using modified TF-IDF and word2vec. PLoS One 15:e0230442. doi: 10.1371/journal.pone.0230442, PMID: 32191738 PMC7081997

[ref36] Nieuwboer M. S. van der Sande R. van der Marck M. A. Olde Rikkert M. G. M. Perry M. (2019). Clinical leadership and integrated primary care: a systematic literature review. Eur. J. Gen. Pract. 25, 7–18. doi: 10.1080/13814788.2018.1515907, PMID: 30474447 PMC6394325

[ref37] Rockstuhl T. Van Dyne L. (2018). A bi-factor theory of the four-factor model of cultural intelligence: meta-analysis and theoretical extensions. Organ. Behav. Hum. Decis. Process. 148, 124–144. doi: 10.1016/j.obhdp.2018.07.005

[ref38] Safari S. Rezaei M. Mohammadi E. (2024). Psychometric properties of the educational leadership scale for nursing students. A psychometric study. Int. J. Nurs. Sci. 11, 205–212. doi: 10.1016/j.ijnss.2023.12.00438707684

[ref39] Smith J. D. Jones A. B. Williams C. E. (2019). Validation of the nursing education leadership assessment scale (NELAS) in European nursing education settings. J. Nurs. Educ. 58, 145–153. doi: 10.3928/01484834-20190215-02

[ref40] Steinert Y. Naismith L. Mann K. (2012). Faculty development initiatives designed to promote leadership in medical education. A BEME systematic review: BEME guide no. 19. Med. Teach. 34, 483–503. doi: 10.3109/0142159X.2012.680937, PMID: 22578043

[ref41] Stephens M. Ormandy P. (2019). An evidence-based approach to measuring affective domain development. J. Prof Nurs 35, 216–223. doi: 10.1016/j.profnurs.2018.12.004, PMID: 31126399

[ref42] Wolf M. G. McNeish D. (2023). Dynamic: an R package for deriving dynamic fit index cutoffs for factor analysis. Multivar. Behav. Res. 58, 189–194. doi: 10.1080/00273171.2022.2163476, PMID: 36787513

[ref43] Wong A. K. C. Chan E. A. Chan K. S. Y. Johnston J. Malik G. Peddle M. . (2024). The effects of video-based simulation in collaborative learning in a student-led global classroom (CLSGC) program on non-technical skills among undergraduate nursing students in three regions: a mixed-methods study. Nurse Educ. Today 143:106381. doi: 10.1016/j.nedt.2024.106381, PMID: 39236596

[ref44] Wu Y. Huang K. Wen S. Xiao B. Feng L. (2022). Validation of the Chinese version of the stigma scale of epilepsy. Front. Neurol. 13:796296. doi: 10.3389/fneur.2022.796296, PMID: 35197923 PMC8858795

[ref45] Xu Y. Zheng Y. Wang H. Huang F. (2024). Development and psychometric properties of clinical learning environment scale for Chinese nursing students. BMC Med. Educ. 24:103. doi: 10.1186/s12909-024-05087-w, PMID: 38297299 PMC10832204

[ref46] Ystaas L. M. K. Nikitara M. Ghobrial S. Latzourakis E. Polychronis G. Constantinou C. S. (2023). The impact of transformational leadership in the nursing work environment and patients' outcomes: a systematic review. Nurs Report 13, 1271–1290. doi: 10.3390/nursrep13030108, PMID: 37755351 PMC10537672

[ref47] Zhang C. Yang Z. Zhang H. (2022). Psychometric evaluation of the Chinese version of occupational LowBack pain prevention Behaviors questionnaire among clinical nurses: a validation study. Front. Public Health 10:827604. doi: 10.3389/fpubh.2022.827604, PMID: 35400039 PMC8984022

