# Supplementary material for: Psychometric evaluation of the Chinese version of the Educational Leadership Scale for Nursing Students: a quantitative cross-sectional design
Source: Front Psychol. 2025 Nov 12;16:1604416. doi: 10.3389/fpsyg.2025.1604416 (PMC12646919; doi:10.3389/fpsyg.2025.1604416)
Supplement: Supplementary file 1 [file Table_1.docx]

**Output of the mean, standard deviation, skewness, and kurtosis results for the 19 items of the scale.**

| **Descriptive Statistics** | | | | | | | |
| --- | --- | --- | --- | --- | --- | --- | --- |
|  | N | Mean | Std.Deviation | Skewness | | Kurtosis | |
|  | Statistic | Statistic | Statistic | Statistic | Std. Error | Statistic | Std. Error |
| Q1 | 508 | 2.42 | 1.25 | 0.65 | 0.11 | -0.57 | 0.22 |
| Q2 | 508 | 2.65 | 1.35 | 0.24 | 0.11 | -1.24 | 0.22 |
| Q3 | 508 | 2.39 | 1.16 | 0.47 | 0.11 | -0.61 | 0.22 |
| Q4 | 508 | 2.4 | 1.22 | 0.70 | 0.11 | -0.41 | 0.22 |
| Q5 | 508 | 2.43 | 1.23 | 0.58 | 0.11 | -0.66 | 0.22 |
| Q6 | 508 | 2.33 | 1.27 | 0.68 | 0.11 | -0.65 | 0.22 |
| Q7 | 508 | 2.36 | 1.19 | 0.64 | 0.11 | -0.47 | 0.22 |
| Q8 | 508 | 2.42 | 1.28 | 0.61 | 0.11 | -0.77 | 0.22 |
| Q9 | 508 | 2.47 | 1.32 | 0.55 | 0.11 | -0.81 | 0.22 |
| Q10 | 508 | 2.34 | 1.25 | 0.55 | 0.11 | -0.78 | 0.22 |
| Q11 | 508 | 2.52 | 1.34 | 0.55 | 0.11 | -0.88 | 0.22 |
| Q12 | 508 | 2.35 | 1.25 | 0.72 | 0.11 | -0.46 | 0.22 |
| Q13 | 508 | 2.49 | 1.21 | 0.49 | 0.11 | -0.61 | 0.22 |
| Q14 | 508 | 2.46 | 1.28 | 0.49 | 0.11 | -0.83 | 0.22 |
| Q15 | 508 | 2.44 | 1.30 | 0.67 | 0.11 | -0.64 | 0.22 |
| Q12 | 508 | 2.5 | 1.21 | 0.28 | 0.11 | -1.00 | 0.22 |
| Q17 | 508 | 2.46 | 1.26 | 0.55 | 0.11 | -0.75 | 0.22 |
| Q18 | 508 | 2.49 | 1.29 | 0.58 | 0.11 | -0.72 | 0.22 |
| Q19 | 508 | 2.53 | 1.24 | 0.56 | 0.11 | -0.56 | 0.22 |
